# Clinico-Epidemiological Profile of Vitiligo Among Patients Attending a Tertiary Care Centre of North-East India

**DOI:** 10.7759/cureus.58804

**Published:** 2024-04-23

**Authors:** Debashree Roy Saha, Somnath Roy, Rashmi Ahmed, Putul Mahanta

**Affiliations:** 1 Dermatology, Nalbari Medical College and Hospital, Nalbari, IND; 2 Internal Medicine, Gauhati Medical College and Hospital, Guwahati, IND; 3 Community Medicine, Lakhimpur Medical College, Lakhimpur, IND; 4 Forensic Medicine and Toxicology, Nalbari Medical College and Hospital, Nalbari, IND

**Keywords:** leukotrichia, koebner's phenomena, autoimmune disease, cutaneous, vitiligo vulgaris

## Abstract

Objectives

Vitiligo is a widespread cutaneous disorder. The present study aims to evaluate the epidemiologic profile of vitiligo and investigate its different clinical forms, disease activity, hereditary associations, triggering factors, and probable association with other diseases.

Methods

This prospective observational study was conducted over one year, from 2019 to 2020, and included 120 cases demonstrating definite clinical evidence of vitiligo. All selected patients underwent a detailed medical history interview. Specific enquiries were made regarding precipitating factors, clinical features of the disease, histories of other autoimmune diseases, and family histories. Thorough clinical, laboratory, and cutaneous examinations were performed on all patients. Descriptive statistical methods and diagrams were used to summarise the data.

Results

The age at presentation (31 patients, 25.8%) and the onset of the disease (32 patients, 26.6%) was predominantly in the second decade of life. The condition was usually progressive, with vitiligo vulgaris being the most prevalent type (56 cases, 46.7%). Disease onset (37 individuals, 30.8%) and the prevalence of lesions were higher in the lower leg. Body surface area involvement was ≤1% in 72 (60.0%) patients. Itching and trauma were the typical initiating factors. Leukotrichia in 38 (31.7%) cases, Koebner's phenomena in 23 (19.1%) cases, and a positive family history in 26 (21.7%) cases were observed. Thyroid dysfunction, hypertension, and various skin conditions are associated with the disease.

Conclusion

Vitiligo is more common in the young population. The condition is often progressive, with vitiligo vulgaris being the most common type. Itching and trauma are frequent initiating factors. Monitoring patients for associated diseases may be crucial for diagnosis and treatment outcomes.

## Introduction

Vitiligo is a widespread cutaneous disorder that causes depigmentation and affects 0.5%-2% of the global population [1]. It is an acquired condition of the skin and hair that progresses over time and is marked by well-defined, milky white macules that lack distinguishable melanocytes. It is recognized as an autoimmune disorder linked to metabolism and oxidative stress, involving cellular detachment disorders, genetic factors, and environmental variables [1,2]. The occurrence of other autoimmune conditions in patients and first-degree relatives supports the autoimmune origin of the condition [3].

Individuals suffering from vitiligo, especially those with darker skin tones, experience a markedly diminished quality of life along with substantial negative effects on their self-esteem and social interactions [4]. Patients often face societal stigma due to widespread preconceived notions, misinformation, taboos, a dearth of scientific assessment, and confusion regarding vitiligo and leprosy [5]. Visible lesions, lesions in sensitive areas, and female sex are documented to be significantly associated with the poor quality of life of the patients [6].

Across studies from India, the prevalence of vitiligo has consistently been reported to be between 0.25%-4% [7]. A recent study from northeastern India documented the rate of occurrence of childhood vitiligo as 2.04% [8]. However, there are not enough detailed studies on the clinical-epidemiological features of vitiligo from the Northeastern region of India. Therefore, the present study was conducted in a tertiary care facility in northeastern India to evaluate the epidemiologic profile of vitiligo and investigate the different clinical forms, disease activity, hereditary associations, triggering factors, and probable association of vitiligo with other diseases.

## Materials and methods

The present descriptive observational study was conducted among patients presenting with vitiligo at the Outpatient Department (OPD) of Dermatology and Sexually Transmitted Diseases (STD), Gauhati Medical College and Hospital, Guwahati (GMCH), from 2019 to 2020. A total of 120 cases showing definite clinical evidence of vitiligo were included in the study. Proper informed consent was obtained from the participants. The Ethics Committee of GMCH approved the study vide Ref: EC/MC/GMC/274.

Patients with typical morphological lesions of vitiligo were included. A clinical diagnosis of vitiligo was made only when there was unequivocal evidence of well-circumscribed, depigmented macules and patches of milky white colour. Patients with an early lesion, a slightly white or hypopigmented macule with an indistinct border, were also included in the study. Cases showing white patches due to secondary causes like chemicals, burns, or other diseases were excluded from this study. Also, cases that had received topical or systemic treatment before attending the Dermatology OPD were excluded from the study.

All selected patients were interviewed for a detailed medical history. The chief complaints were recorded including age, initial site of onset, duration of the disease, history of spontaneous re-pigmentation, and any associated cutaneous or systemic illnesses. A specific enquiry was made regarding the precipitating/initiating factors, the disease's activity, the presence of photophobia, night blindness, and any hearing defect. The history of other autoimmune diseases and family history was probed in detail, noting the family history, if present, and the exact relation with the patient.

All patients underwent a detailed clinical examination. In each case, a thorough general physical examination, and examinations of the cardio-respiratory system, central and peripheral nervous systems, musculoskeletal system, and abdominal area were performed.

Documentation of cutaneous findings included assessment of the body surface area involved, location and distribution of lesions, classification of the type of vitiligo, presence of Koebner's phenomenon, presence of leucotrichia, and presence of any other associated dermatological conditions such as halo nevi, alopecia areata, psoriasis, atopic dermatitis, etc. The body surface area involved in the vitiliginous process was calculated using the Rule of Nines.

Patients were examined in the Departments of Ophthalmology and Otorhinolaryngology of GMCH to study the possible involvement of the eyes and cochlear organs. The ophthalmological workup included a careful slit-lamp examination and fundoscopy, with special attention given to colour alteration of the iris as well as alteration in the pigment epithelium of the choroids. The audiological examination included an air-bone conduction test and a pure tone audiometry test of both ears. Routine examinations of blood, urine, and stool, peripheral blood smear study, liver function tests, blood sugar estimation, thyroid profile, and other investigations were conducted wherever necessary.

Statistical analysis

The tabulation and analysis of data were conducted using Microsoft Excel (Microsoft Corporation, Redmond, Washington) and IBM SPSS Statistics for Windows, Version 23 (Released 2015; IBM Corp., Armonk, New York). To summarize the data, descriptive statistics were used. Categorical data were expressed as counts and percentages, while continuous data were presented as range, mean, and standard deviation. Data on precipitating factors of vitiligo, distribution of lesions, and other associated cutaneous and non-cutaneous conditions, which might contain one or more responses per respondent, were represented as bar diagrams.

## Results

Vitiligo accounted for an annual incidence of 1.12% of all dermatological appointments during the study period. Out of 120 vitiligo cases in the study, the male-to-female ratio was almost equal, with 61 (50.8%) females and 59 (49.2%) males. The patients' ages ranged from 4 to 81 years, with a mean age at presentation (± standard deviation) of 28.8 years (± 17.9 years). The maximum number of patients were in the age group 10-19 years (31 patients, 25.8%), followed by those aged 20-29 years. The mean age at onset (± standard deviation) was 25.1 years (±16.5 years). Most cases had their disease onset in the second decade of life (n = 32, 26.7%). The duration of the disease was less than six months in 30 patients (25.0%), while it ranged from one to five years in one-third of the patients (n = 40, 33.3%). The mode of onset was unicentric in 94.2% (113/120) of patients. The lower extremities were the primarily involved initial site in 37 patients (30.8%), followed by the face in 27 patients (22.5%), the trunk in 20 patients (16.7%), and the upper limb in 13 patients (10.8%), as shown in Table 1.

**Table 1:** Characteristics of the study participants The data are presented as the frequency of patients (n) and percentage (%)

Variables	Sub-groups	Female, n=61	Male, n=59	Total, n=120
Age at presentation	0-9	8 (6.7%)	4 (3.3%)	12 (10.0%)
	10-19	17 (14.2%)	14 (11.7%)	31 (25.8%)
	20-29	17 (14.2%)	9 (7.5%)	26 (21.7%)
	30-39	7 (5.8%)	14 (11.7%)	21 (17.5%)
	40-49	5 (4.2%)	7 (5.8%)	12 (10.0%)
	50-59	5 (4.2%)	4 (3.3%)	9 (7.5%)
	≥60	2 (1.7%)	7 (5.8%)	9 (7.5%)
Age of onset	0-9	12 (10.0%)	10 (8.3%)	22 (18.3%)
	10-19	16 (13.3%)	16 (13.3%)	32 (26.6%)
	20-29	16 (13.3%)	5 (4.2%)	21 (17.5%)
	30-39	7 (5.8%)	16 (13.3%)	23 (19.1%)
	40-49	4 (3.3%)	4 (3.3%)	8 (6.6%)
	50-59	5 (4.2%)	6 (5.0%)	11 (9.2%)
	≥60	1 (0.8%)	2 (1.7%)	3 (2.5%)
Duration of the disease	<6 months	17 (14.2%)	13 (10.8%)	30 (25.0%)
	6 months to 1 year	14 (11.7%)	15 (12.5%)	29 (24.2%)
	>1 year to 5 years	25 (20.8%)	15 (12.5%)	40 (33.3%)
	>5 to 10 years	1 (0.8%)	4 (3.3%)	5 (4.7%)
	>10 years	4 (3.3%)	12 (10.0%)	16 (13.3%)
Mode of onset	Unicentric	57 (47.5%)	56 (46.7%)	113 (94.2%)
	Multicentric	4 (3.3%)	3 (2.5%)	7 (5.8%)
Sites of onset	Face	15 (12.5%)	12 (10.0%)	27 (22.5%)
	Neck	6 (5.0%)	2 (1.7%)	8 (6.7%)
	Trunk	7 (5.8%)	11 (9.2%)	18 (15.0%)
	Scalp	3 (2.5%)	3 (2.5%)	6 (5.0%)
	Upper Limb	7 (5.8%)	6 (5.0%)	13 (10.8%)
	Lower Leg	16 (13.3%)	21 (17.5%)	37 (30.8%)
	Genitalia	3 (2.5%)	1 (0.8%)	4 (3.3%)
	Face and lower leg	4 (3.3%)	2 (1.7%)	6 (5.0%)

**Figure 1:**
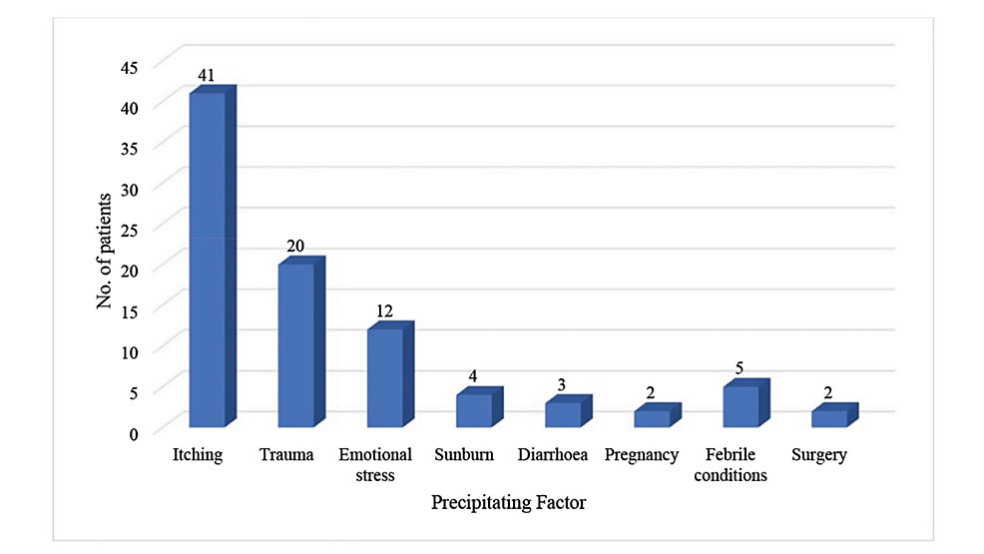
Precipitating factors of vitiligo The data are presented as the frequency (n) of patients with each precipitating factor

In the present study, 80 patients (66.7%) reported a history of one or more precipitating or initiating factors, either local or systemic, before the evolution of typical vitiligo lesions. The most common precipitating factor was itching at the site of future lesions in 41 patients (34.2%), followed by blunt trauma in 20 patients (16.7%) and emotional stress in 12 patients (10.0%) (Figure 1).

The disease was progressive in 107 patients (89.2%), out of which 81 patients (67.5%) had slow progression, and eight patients (6.6%) had rapid progression. Meanwhile, 18 patients (15.0%) with initially stable disease developed rapid or slow progression. Spontaneous re-pigmentation was observed in 16 patients (13.3%). The generalized form of the disease was observed in the majority of 76 patients (63.3%), with vitiligo vulgaris being the most typical type (56 patients, 46.7%). Among the 44 cases (36.7%) with the localized form of the disease, focal vitiligo was mainly observed. Leukotrichia was present in 38 patients (31.7%). Hair depigmentation was typically observed over the legs in 15 patients (12.5%), followed by the scalp in eight patients (6.7%), the chest in four patients (3.3%), and the eyebrows and eyelids in seven patients (5.8%). Koebner's phenomenon was observed in 23 patients (19.1%). These lesions were mainly located on the traumatic sites such as knuckles, elbows, knees, legs, and forearms, and they roughly resembled the size of the original traumatic lesions. The body surface area involved in the vitiliginous process was calculated using the Rule of Nines. The minimum body surface area involved was 0.07%, and the maximum body surface area involved was 92%. Of the 120 patients, 119 (99.2%) had less than 20% of body surface area involvement. Body surface area involvement was 1% or below in 72 patients (60%), as shown in Table 2.

**Table 2:** Clinical features and body involvement pattern of vitiligo The data are presented as the frequency of patients (n) and percentage (%) BSA: body surface area

Disease characteristics	Female, n=61	Male, n=59	Total, n=120
Nature of disease			
Stable	8 (6.6%)	5 (4.2%)	13 (10.8%)
Stable initially, progressive rapidly later on	4 (3.3%)	2 (1.7%)	6 (5.0%)
Stable initially, progressive slowly later on	4 (3.3%)	8 (6.6%)	12 (10.0%)
Progressive rapidly	4 (3.3%)	4 (3.3%)	8 (6.6%)
Progressive slowly	41 (34.2%)	40 (33.3%)	81 (67.5%)
Spontaneous re-pigmentation	6 (5.0%)	10 (8.3%)	16 (13.3%)
Type of vitiligo			
vitiligo vulgaris	23 (19.2%)	33 (27.5%)	56 (46.7%)
vitiligo acro facialis	12 (10.0%)	7 (5.8%)	19 (15.8%)
vitiligo universalis	1 (8.3%)	0	1 (8.3%)
Focal	14 (11.7%)	9 (7.5%)	23 (19.2%)
Mucosal	7 (5.8%)	3 (2.5%)	10 (8.3%)
Segmental	4 (3.3%)	7 (5.8%)	11 (9.2%)
Leukotrichia	14 (11.7%)	24 (20.0%)	38 (31.7%)
Koebner's phenomenon	8 (6.6%)	15 (12.5%)	23 (19.1%)
BSA involved			
0-1	44 (36.7%)	28 (23.3%)	72 (60.0%)
>1-5	15 (12.5%)	18 (15.0%)	33 (27.5%)
>5-10	1 (8.3%)	11 (9.2%)	12 (10.0%)
>10-50	0	2 (1.7%)	2 (1.7%)
>50	1 (8.3%)	0	1 (8.3%)

**Figure 2:**
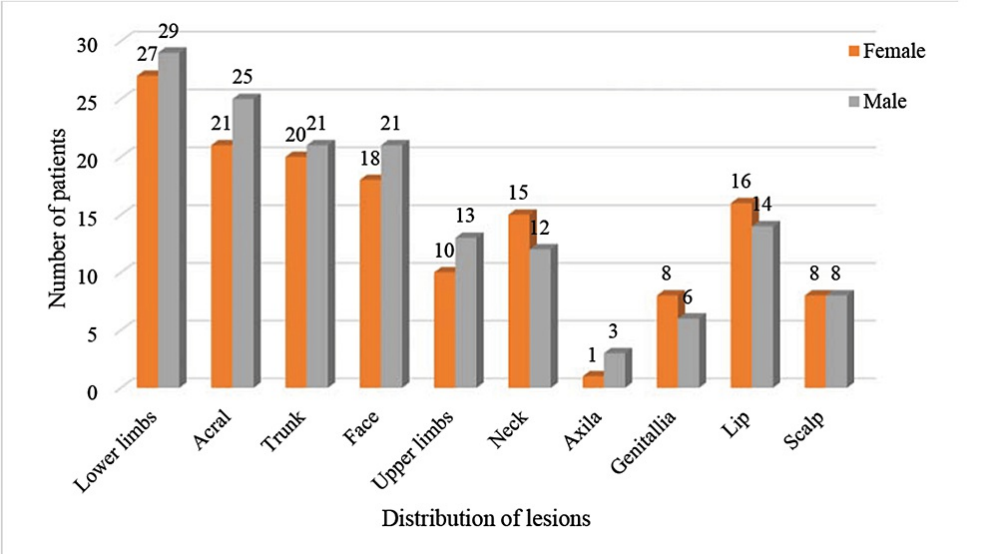
Distribution of lesions The data are presented as the frequency of lesions (n)

As seen in Figure 2, the extensor part of the lower limbs accounted for the maximum number of lesions, with 56 (20.3%) out of 276 total lesions, followed by the acral (16.7%), truncal (14.8%), and facial regions (14.1%). Lesions were less frequent over the genital mucosa, with 14 cases (5.0%).

Among the study participants, 26 (21.7%) patients had a family history of vitiligo. Of these, the majority, 12 cases (10.0%), had one or more first-degree relatives with the disease, and 10 cases (8.3%) had affected first cousins (Table 3).

**Table 3:** Family history of vitiligo The data are presented as the frequency of patients (n) and percentage (%)

Family history of vitiligo	No. of patients, n=120
None	94 (78.3%)
First degree relative	12 (10.0%)
Second degree relative	4 (3.3%)
Third-degree relatives	10 (8.3%)

**Figure 3:**
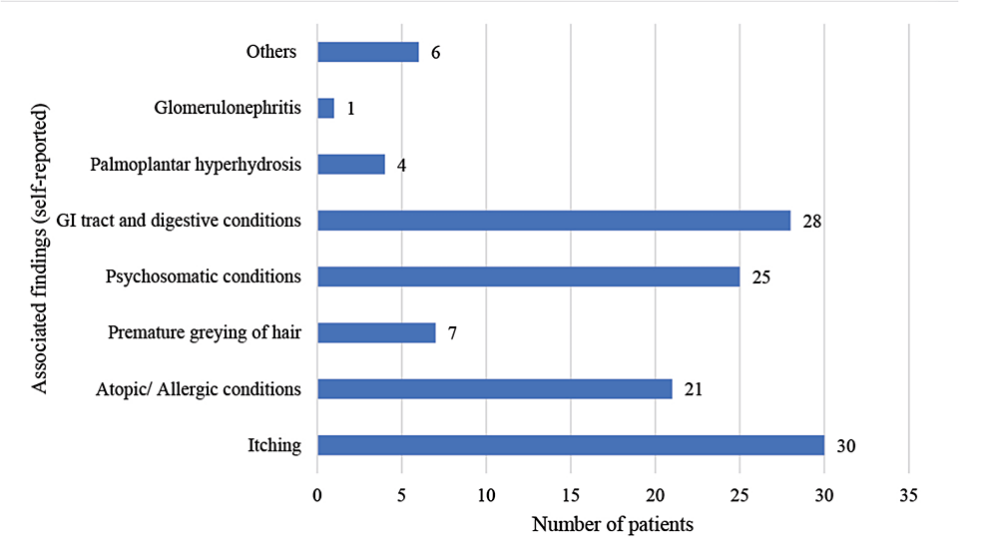
Associated conditions (self-reported) The data are presented as the frequency of patients (n) with self-reported associated conditions

Thirty patients (25%) reported itching as the most common associated finding. Additionally, patients reported various digestive conditions (28 patients; 23.3%), atopic conditions, particularly allergic rhinitis (21 patients; 17.5%), and psychosomatic disorders (25 patients; 20.8%) as typical findings. Seven patients reported premature greying of hair (Figure 3).

**Figure 4:**
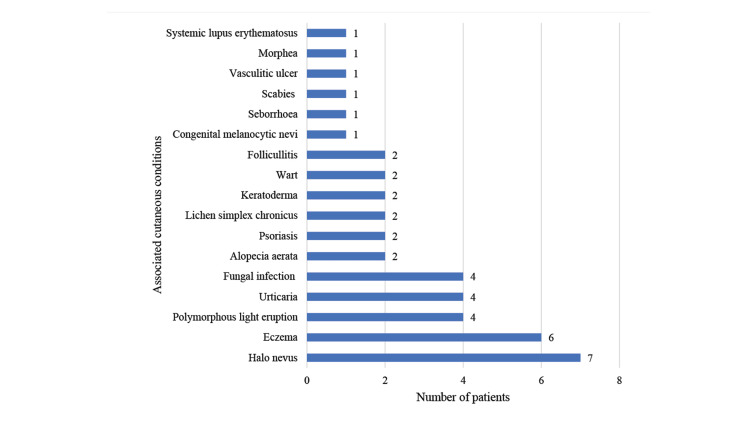
Associated cutaneous conditions The data are presented as the frequency of patients (n) with different cutaneous conditions

The cutaneous examinations revealed that seven patients (5.8%) had associated halo nevi, among whom four patients had more than one halo nevus. Eczematous lesions, urticarial lesions, polymorphous light eruption lesions, and fungal infections were the main cutaneous conditions observed among the patients. One patient (0.8%) in our study had a morphea lesion over the abdomen and back, while another had vitiligo with systemic lupus erythematosus (SLE) (Figure 4).

In the general examination, pallor was observed in nine (7.5%) patients; hypertension was found in seven (5.8%), while 10 (8.3%) patients were found with irregular heart rates. Out of 120 patients, 10 (8.3%) had thyroid disease, of which eight (6.7%) had hypothyroidism, and two patients (1.7%) had hyperthyroidism. Autoantibodies were detected in four out of the eight cases with hypothyroidism and in both cases with hyperthyroidism. Diabetes mellitus was found in two (1.7%) patients. In 19 patients (15.8%), blood haemoglobin level was less than 10 g/dL, with the lowest being 5 g/dL. Peripheral blood smears showing features of macrocytosis, anisocytosis, poikilocytosis, and macro-ovalocytes were present in two patients (1.7%), consistent with the diagnosis of pernicious anaemia. Routine examination of stool revealed *E. histolytica *in eight patients (6.7%), Giardia in one patient, and roundworm in one patient, accounting for 8.3% of cases (Table 4).

**Table 4:** Associated findings on general and laboratory investigation of the 120 vitiligo patients The data are presented as the frequency of patients (n) and percentage (%) PBS: peripheral blood smear, Hb: haemoglobin, SGOT: serum glutamic-oxaloacetic transaminase, SGPT: serum glutamic-pyruvic transaminase, R/E: routine examination

Associated findings on general and laboratory investigation	No. of patients
Pallor	9 (7.5%)
Hypertension	7 (5.8%)
Bradycardia	5 (4.2%)
Tachycardia	5 (4.2%)
Thyroid dysfunction	10 (8.3%)
Anaemia (Hb < 10 g/dL)	19 (15.8%)
Pernicious anaemia from PBS	2 (1.7%)
Elevated blood sugar	2 (1.7%)
Elevated SGOT	3 (2.5%)
Elevated SGPT	4 (3.3%)
Parasitic infection in stool R/E	10 (8.3%)

Audiological investigation of the patients revealed that 23 patients (19.2%) had hypoacusis, among whom 12 were in the age group of 5-30 years and 11 were in the age group of 31-60 years. Hypoacusis was of the sensorineural type in 20 patients (16.7%) and of the conductive type in three patients (2.5%). Eighteen patients (15.0%) were found to have ocular abnormalities on ophthalmological examination, among which 14 patients had generalized vitiligo and four had localized vitiligo. The spectrum of ocular manifestations observed in vitiligo patients included hypopigmented patches in pairs (three patients), heterochromic iris and retinal pigment epithelium (two patients), atrophy and hypopigmentation (four patients), hypopigmented patches (three patients), and uveitis (anterior: 3; posterior: 3). One patient had features suggestive of Vogt-Koyanagi-Harada syndrome with diffuse alopecia over the scalp, depigmentation of scalp hair, headache, neck stiffness, and difficulty in hearing and vision.

## Discussion

Vitiligo is an acquired autoimmune condition of the skin that causes depigmentation, which can be progressive throughout the patient's lifespan and significantly affect the sufferer's quality of life. The ethnic, geographical, and environmental diversity contributes to the broad range of vitiligo prevalence in India. The clinico-epidemiological profile of the disease is less documented in the northeastern region of India. The current study evaluates vitiligo's epidemiological and clinical profile among patients attending the Outpatient Department of Dermatology and STD, Gauhati Medical College and Hospital, Guwahati.

Of 120 patients, 59 (49.2%) were males and 61 (50.8%) were females. Similar to our findings, vitiligo is documented to affect both genders almost equally. However, instances of a female majority in some studies may be attributed to their better health-seeking behaviour due to aesthetic concerns [7,9-11]. Most cases have their age at presentation and onset of the disease in the second decade of life, which agrees with other studies [11,12]. The disease duration was 1-5 years in one-third of the patients (33.3%), corresponding to a similar study from Gujarat [13]. Most patients reporting a longer duration of the disease at presentation could be due to opting for alternative medication before seeking medical advice from a specialized centre. The unicentric mode of onset was observed in more than 90% of patients, with lower extremities, face, and trunk being the most involved initial sites. The findings are in agreement with similar studies [7,11].

The most typical precipitating factors were itching, trauma, and emotional stress. The disease was progressive in most cases (89.2%), with vitiligo vulgaris being the most typical type (56 patients, 46.7%), which agrees with the findings of Vora et al. [13]. Leukotrichia was present in 38 patients (31.7%) and was typically observed over the legs and scalp in our study. The incidence of leukotrichia has been reported to range from 9 to 48.4% in vitiligo patients and is generally regarded as a predictor of poor outcomes [14]. Spontaneous re-pigmentation was observed in 16 (13.3%) patients. Koebner's phenomenon was observed in 23 patients (19.1%), agreeing with another study [13]. Of the 120 patients, 119 (99.2%) had less than 20% of body surface area involvement. Family histories are significant in the occurrence of vitiligo. A family history of vitiligo was present in 26 (21.7%) patients. The findings are in concordance with other similar research [13].

The most common associated finding was itching, reported by 30 patients (25%), among whom four were found to have eczematous lesions, four had urticarial lesions, four had polymorphous light eruption (PLE) lesions, one had a scabetic lesion, and the remaining 17 patients experienced chronic pruritus with no lesions other than scratch marks. Atopic conditions, particularly allergic rhinitis (17.5%), and psychosomatic disorders (20.8%), were typically associated with findings among the patients. Increased prevalences of atopic illness in vitiligo have been previously documented [15].

Vitiligo is linked to several systemic and cutaneous illnesses. A recent report documented a significant frequency of autoimmune comorbidities in patients with vitiligo [3]. In the present study, 10 out of 120 patients (8.3%) had thyroid disease. The cutaneous examinations revealed that seven (5.8%) patients had associated halo nevus. A halo nevus in 1% to 35% of vitiligo patients is regarded as a clinical marker for signalling the onset of vitiligo, particularly among children [7]. Pernicious anaemia was observed in two (1.7%) patients. One patient (0.83%) had presented with morphea of 10 months' duration over the abdomen and back, and vitiliginous lesions of two years' duration over the scalp and retroauricular area. ANA was positive in the patient with a significant rise in titre. Another patient had presented with features of systemic lupus erythematosus for two months, having vitiliginous lesions for eight years. ANA and anti-ds DNA were present at a significant titre in the patient.

A recent meta-analysis reported that patients with vitiligo had six times higher odds of sensorineural hearing loss [16]. In our study, 23 patients (19.2%) had hypoacusis, of which hypoacusis was of the sensorineural type in 20 patients. Vitiligo is a systemic illness that affects melanocyte function in organs other than the skin, such as the eyes and ears. Ear melanocytes play a role in the auditory process; hence, their damage may result in hearing loss [17]. Eighteen patients (15.0%) were found to have ocular abnormalities on ophthalmological examination. Although ocular melanocytes do not directly contribute to the detection or transfer of visual information, vitiligo may still be associated with ocular abnormalities [17].

Limitation

The present study was conducted in a single tertiary care centre over one year. Long-term studies with a larger sample size will further help to better understand the clinico-epidemiological aspects of the disease.

## Conclusions

Vitiligo is primarily distributed among the young population, regardless of gender, in the study region. The disease is predominantly progressive, with vitiligo vulgaris being the most prevalent type. Itching and trauma are the typical initiating factors. The disease is associated with other autoimmune diseases and cutaneous conditions.

The genesis and pathophysiology of vitiligo remain challenging despite substantial ongoing advances in our understanding of this complex skin disorder. The frequent association of vitiligo with other autoimmune diseases and the presence of ocular and audiologic abnormalities suggest that vitiligo is more than just a skin disease and thus necessitates thorough screening of all patients. Screening individuals with associated disorders may be crucial for facilitating better diagnosis and treatment of the disease.
